# Colchicine to Reduce Atrial Fibrillation in the Postoperative Period of Myocardial Revascularization

**DOI:** 10.5935/abc.20160082

**Published:** 2016-07

**Authors:** Camila Stuchi Zarpelon, Miguel Chomiski Netto, José Carlos Moura Jorge, Cátia Carolina Fabris, Dieli Desengrini, Mariana da Silva Jardim, Diego Guedes da Silva

**Affiliations:** Irmandade da Santa Casa de Misericórdia de Curitiba, Curitiba, PR - Brazil

**Keywords:** Colchicine/therapeutic use, Atrial Fibrillation/mortality, Myocardial Revascularization, Postoperative Period

## Abstract

**Background:**

The high prevalence of atrial fibrillation (AF) in the postoperative period of myocardial revascularization surgery increases morbidity and mortality.

**Objective:**

To assess the efficacy of colchicine to prevent AF in the postoperative period of myocardial revascularization surgery, the impact of AF on hospital length of stay and death, and to identify its risk factors.

**Methods:**

Between May 2012 and November 2013, 140 patients submitted to myocardial revascularization surgery were randomized, 69 to the control group and 71 to the colchicine group. Colchicine was used at the dose of 1 mg orally, twice daily, preoperatively, and of 0.5 mg, twice daily, until hospital discharge. A single dose of 1 mg was administered to those admitted 12 hours or less before surgery.

**Results:**

The primary endpoint was AF rate in the postoperative period of myocardial revascularization surgery. Colchicine group patients showed no reduction in AF incidence as compared to control group patients (7.04% versus 13.04%, respectively; p = 0.271). There was no statistically significant difference between the groups regarding death from any cause rate (5.6% versus 10.1%; p = 0,363) and hospital length of stay (14.5 ± 11.5 versus 13.3 ± 9.4 days; p = 0.490). However, colchicine group patients had a higher infection rate (26.8% versus 8.7%; p = 0.007).

**Conclusion:**

The use of colchicine to prevent AF after myocardial revascularization surgery was not effective in the present study. Brazilian Registry of Clinical Trials number RBR-556dhr.

## Introduction

Atrial fibrillation in the postoperative period of myocardial revascularization surgery (AF-POMR) occurs in 10% to 65% of patients,^1-5^ increasing morbidity and mortality after surgery.^6,7^ It is associated with an increase in hospital length of stay, and, thus, in costs,^6,8,9^ and can cause serious clinical complications, such as hypotension, heart failure, stroke and other thromboembolic disorders.^10^

Although not completely understood, the electrophysiological mechanism of AF-POMR is believed to be reentry. Some complications inherent in the postoperative period can be the trigger in patients predisposed to AF. Perioperative atrial trauma, pericardial inflammatory process secondary to surgical manipulation, autonomic disorder and plasma volume changes are some predisposing factors.^6,11^

Colchicine is the classic drug to treat gout, in addition to being part of the management of Mediterranean fever and Behcet's disease. It is usually classified as an anti-inflammatory agent, although its mechanism of action does not involve the arachidonic acid metabolic pathway. Colchicine binds to non-polymerized tubulin, forming a stable complex that effectively inhibits the dynamic of microtubules, depolymerizing them. Thus, any process requiring changes in the cell cytoskeleton, such as cellular mitosis, exocytosis and neutrophil motility, is affected.^12^ In addition, colchicine has an important effect on atrial myocytes, changing the atrial response to autonomic effects (reducing the sympathetic activity and increasing the parasympathetic one).^13,14^

A COPPS substudy, published by Imazio et al.,^6^ has assessed the use of colchicine to prevent atrial fibrillation (AF) in the postoperative period of cardiac surgery. A reduction from 22% to 12% was observed in the AF rate as compared to the control group (p = 0.021), in addition to a reduction in hospital length of stay (p = 0.04) and rehabilitation time (p = 0.009)_._ However, in that study, colchicine was initiated only on the third postoperative day; however, the highest AF incidence occurs on the first 2 to 3 days after cardiac intervention.^15^

This study aimed at assessing colchicine to prevent AF in patients undergoing myocardial revascularization surgery.

## Methods

### Study Design and Participants

This is a prospective, randomized, open, single-center clinical assay, whose 140 participants were recruited from the Hospital Santa Casa de Misericórdia de Curitiba.

This study followed the Declaration of Helsinki, which provides recommendations to biomedical research involving human beings, and was submitted to the Committee of Ethics in Research with Human Beings of the Pontifícia Universidade Católica do Paraná (PUC-PR) (protocol 50883).

### Inclusion Criteria

Patients willing and able to provide informed consent term were recruited. The inclusion criteria were as follows: minimum age of 18 years; indication for elective myocardial revascularization surgery; and sinus rhythm on the day prior to surgery. Mean ages were 60.3 ± 8.1 years and 61.5 ± 10.3 years in the control and colchicine groups (p = 0.44), respectively, and 45 participants were of the female sex, and 95, of the male sex.

### Exclusion Criteria

The exclusion criteria were: contraindication to the study medication; previously diagnosed AF or atrial flutter; need for heart valve surgery associated; severe liver disease (aminotransferase levels increased more than 1.5-fold the normal value); renal failure (creatinine > 2 mg/dL); known gastrointestinal diseases; current colchicine treatment; cardiogenic shock; severe arrhythmias; neoplasms; non-communicative patients; simultaneous use of antiarrhythmic drugs, except for digoxin, beta-blockers and calcium channel blockers.

### Intervention

From May 2012 to November 2013, 140 patients submitted to myocardial revascularization surgery were randomized into two groups: control group, not receiving the study medication; and therapeutic group, medicated with colchicine. The therapeutic group received oral colchicine at the dose of 1 mg, twice daily, in the preoperative period (initiated 24 hours before surgery), followed by 0.5 mg, orally or nasogastrically, twice daily, until hospital discharge. If the patient was admitted only 12 hours before surgery, colchicine was administered orally, at a single dose of 1 mg, the night before surgery. Clinical management and medications routinely used in both groups were not changed.

The cardiac rhythm was defined as AF when no P wave was detected before the QRS complex, and the heart rate was irregular. To be considered in this study, AF episodes should last at least 5 minutes or, if shorter, they should have caused hemodynamic instability. Atrial fibrillation was identified via electrocardiography by using continuous cardiac monitoring and 12-lead electrocardiogram (ECG) during intensive care unit (ICU) stay. After ICU discharge, only daily record of 12-lead ECG.

### Endpoints

The primary study endpoint was the AF-POMR rate in the colchicine and control groups. Additional analyses included death from any cause, hospital length of stay and postoperative infection.

### Randomization

Patients were randomly assigned to the groups by use of a computer program (link: http://stattrek.com/statistics/random-number-generator.aspx). Both participants and researchers were instructed on the treatment. Data were collected by use of notification forms, manually completed by study researchers.

### Statistical Analysis

This study required 140 patients (69 in the control group and 71 in the intervention group) to detect AF rates of 20% and 6%, respectively, with an 80% power and p = 0.05 in both groups. The AF rate was estimated for both groups based on the results of a randomized, placebo-controlled study, in which the AF rate was significantly lower in the colchicine group than in the placebo group (12% versus 22%, respectively, p = 0.021).^6^

Quantitative variables were described as means, medians, minimum and maximum values, and standard deviations. Qualitative variables were described as frequencies and percentages. To compare the two groups regarding quantitative variables, Student *t* test or nonparametric Mann-Whitney test was used for independent samples. Regarding qualitative variables, the groups were compared by using Fisher exact test or chi-square test. To compare the two groups regarding time free from AF, log-rank test was used. Values of p < 0.05 indicated statistical significance. Data were assessed by using the Statistica software, v.8.0.

## Results

### Patients' Characteristics

The patients' baseline characteristics were similar in both groups and are shown in Table 1.

**Table 1:** Baseline characteristics of the patients of this study

Characteristic	Colchicine (n = 71)	Control (n = 69)	p
Mean age (mean ± SD), years	61.5 ± 10.3	60.3 ± 8.1	0.44
Age < 65 years (n = 96), n (%)	43 (60.6)	53 (76.8)	
Age > 65 years (n = 44), n (%)	28 (39.4)	16 (23.2)	
Female sex (n = 45), n (%)	22 (30.9)	23 (33.3)	
Male sex (n = 95), n (%)	49 (69.0)	46 (66.7)	
**Previous pathologies, n (%)**			
SA (n = 10)	3 (4.2)	7 (10.1)	
UA (n = 63)	32 (45.1)	31 (44.9)	
CAD (n = 35)	21 (29.6)	14 (20.3)	
AMI (n = 32)	15 (21.1)	17 (24.6)	
SAH (n = 16)	63 (88.7)	61 (88.4)	1.00
Smoking habit (n = 57)	23 (32.4)	34 (49.3)	0.06
Dyslipidemia (n = 88)	44 (61.9)	44 (63.8)	0.86
DM (n = 72)	42 (59.2)	30 (43.5)	0.09
Previous AMI (n = 31)	17 (23.9)	14 (20.3)	0.69
Previous PCI (n = 20)	11 (15.5)	9 (13.1)	0.81
Previous MR (n = 5)	3 (4.2)	2 (2.9)	1.00
FH-CAD (n = 50)	23 (32.4)	27 (39.1)	0.48
Beta-blocker (n = 72)	35 (50.0)	37 (53.6)	0.74
ACEI (n = 63)	33 (46.5)	30 (43.5)	0.74
ARB (n = 22)	12 (16.9)	10 (14.5)	0.82

SA: stable angina; UA: unstable angina; CAD: coronary artery disease; AMI: acute myocardial infarction; SAH: systemic arterial hypertension; DM: diabetes mellitus; PCI: percutaneous coronary intervention; MR: myocardial revascularization; FH: family history; ACEI: angiotensin-converting-enzyme inhibitor; ARB: angiotensin-receptor-blocker.

### Final Result

To assess the primary endpoint (AF-POMR in the colchicine and control groups), AF-POMR episodes from the first postoperative day onward were considered. The colchicine group showed no statistically significant reduction in AF incidence as compared to the control group (7.0% versus 13.0%; p = 0.271; colchicine and control, respectively). After beginning the intervention, the numbers of AF-POMR events in the colchicine and control groups were 5 of 71 versus 9 of 69, respectively (Tabele 2).

**Table 2:** Endpoints of this study according to the colchicine and control groups

Event	Colchicine	Control	p	RRR, %
	(n = 71)	(n = 69)		(95% CI)
**Primary endpoint **				
AF-POMR, %*	7.0	13.0	0.271	46 (-53 a 81)
**Additional analyses**				
Death from any cause, n (%)	4 (5.6)	7 (10.1)	0.363	
Hospital length of stay, days	14.5 ± 11.5	13.3 ± 9.4	0.490	
Postoperative infection, n (%)	19 (26.8)	6 (8.7)	0.007	

RRR: reduction in relative risk with colchicine; CI: confidence interval; AF-POMR: atrial fibrillation in the postoperative period of myocardial revascularization surgery.

* Calculated with Fisher exact test.

The colchicine and control groups showed no statistically significant difference regarding the death from any cause rate (5.6% versus 10.1%, respectively; p = 0.363) and hospital length of stay (14.5 ± 11.5 versus 13.3 ± 9.4 days, respectively; p = 0,490). The colchicine group, however, had a higher infection rate (26.8% versus 8.7%; p = 0.007) (Table 2).

The following clinical characteristics were identified in patients with AF-POMR in both control and colchicine groups as compared to patients without AF-POMR (Table 3): coronary care unit length of stay (7.6 ± 9.0 days versus 3.6 ± 4.6 days, respectively; p = 0.008); use of extracorporeal circulation (ECC) during surgery (28.6% versus 39.7%, respectively; p = 0.566); postoperative beta-blocker use (100.0% versus 93.7%, respectively; p = 1.000); and use of blood products (21.4% versus 26.2%, respectively; p = 1.000). Patients with AF-POMR had a longer coronary care unit length of stay and no other significantly different characteristic.

**Table 3:** Comparison of the clinical characteristics between patients with and without atrial fibrillation (AF) in the postoperative period of myocardial revascularization surgery in the control and colchicine groups

Characteristics	AF, n (%)		
	No (n = 126)	Yes (n = 14)	p
Mean age, years	60.4 ± 9.2	65.6±9.1	0.048
Age < 65 years (n = 96)	88 (69.8)	8 (57.1)	
Age > 65 years (n = 44)	38 (30.2)	6 (42.9)	
Female sex (n = 45)	41 (32.5)	4 (28.6)	
Male sex (n = 95)	85 (67.5)	10 (71.4)	
SAH (n = 124)	114 (90.5)	10 (71.4)	0.057
Previous MR (n = 5)	5 (3.9)	0 (0.0)	1.000
DM (n = 72)	65 (51.6)	7 (50.0)	1.000
CCU length of stay, days	3.6 ± 4.6	7.6 ± 9.0	0.008
ECC (n = 54)	50 (39.7)	4 (28.6)	0.566
Perioperative beta-blocker (n = 132)	118 (93.7)	14 (100.0)	1.000
Use of blood products (n = 36)	33 (26.2)	3 (21.4)	1.000

SAH: systemic arterial hypertension; MR: myocardial revascularization surgery; DM: diabetes mellitus; CCU: coronary care unit; ECC: extracorporeal circulation.

## Discussion

The COPPS-POAF substudy has shown colchicine to be safe and effective to prevent pericarditis and AF-POMR, reducing the incidence of AF (p = 0.021).^6^ The present study showed no statistically significant reduction in the AF-POMR rate, and one cause of that might have been the reduced sample size.

In addition, the cardiac rhythm recording method (continuous cardiac monitoring and 12-lead ECG) might have missed some AF episodes, because the monitors used did not allow data storage for later analysis. The AF-POMR episodes were recorded only when they caused symptoms or hemodynamic change, or, if asymptomatic, when witnessed by an attending physician.

The infection rate of the colchicine group was greater than that of the control group. There is neither a randomized study nor a case report confirming the relationship between colchicine use at therapeutic doses and the increase in the number of infection cases. Our finding might have been random and related to the reduced number of patients, which can be assessed in future studies.

Patients treated with colchicine remain hospitalized longer. That might not be related to the drug itself, but rather to the infection rate, which, as previously stated, was higher in the colchicine group. In addition, the COPPS-POAF substudy, assessing a larger sample, has reported a reduction in the hospital length of stay in the colchicine group as compared to that in the control group (9.4 ± 3.7 versus 10.3 ± 4.3 days; p = 0.040).^6^

Of the 140 patients studied, 54 patients underwent myocardial revascularization surgery with ECC, and 86 patients, without ECC. Hashemzadeh et al.,^16^ assessing 939 patients submitted to myocardial revascularization surgery, have reported AF-POMR in 38 patients of the non-ECC group and in 93 patients of the ECC group (9.8% versus 16.7%, respectively, p=0.002), showing an increase in the AF-POMR rate of patients undergoing surgery with ECC. In the present study, most surgeries were performed without ECC, which might explain the low AF-POMR rate found.

Similarly to the COPPS-POAF substudy, the incidence of death from any cause was not significantly smaller in the colchicine group of this study.^6^

Colchicine is a potent anti-inflammatory drug classically used to treat pericarditis.^12^ Further studies are required to confirm its real safety and efficacy in preventing AF-POMR.

## Conclusion

The use of colchicine to prevent AF-POMR showed no efficacy in the present study.

The authors deny any conflict of interest.
